# Factors associated with balance impairments amongst stroke survivors in northern Benin: A cross-sectional study

**DOI:** 10.4102/sajp.v77i1.1559

**Published:** 2021-09-02

**Authors:** Oyéné Kossi, Mendinatou Agbetou, Sènadé I. Noukpo, Lisa T. Triccas, Daniel-Eude Dossou-Yovo, Elogni R. Amanzonwe, Thierry Adoukonou

**Affiliations:** 1 National School of Public Health and Epidemiological Surveillance (ENATSE), University of Parakou, Parakou, Benin; 2 Unit of Neuro Rehabilitation, Department of Neurology, University Hospital of Parakou, Parakou, Benin; 3 REVAL, Rehabilitation Research Center, Faculty of Rehabilitation Sciences, Hasselt University, Hasselt, Belgium; 4 Department of Neurology, Faculty of Medicine, University of Parakou, Parakou, Benin

**Keywords:** stroke, balance, risk of fall, impairments, rehabilitation

## Abstract

**Background:**

Balance impairment is the predominant risk factor for falls in stroke survivors. A fear of falling after stroke can contribute to sedentary lifestyles, increased disability and risk of recurrence, leading to poor quality of life.

**Objective:**

To determine the frequency and factors associated with balance impairments amongst stroke survivors at the University Hospital of Parakou.

**Method:**

This cross-sectional study included adult stroke survivors. Stroke survivors after discharge were enrolled at the University Hospital of Parakou between 01 January 2020 and 30 September 2020. Balance impairments were measured by using the Berg Balance Scale (BBS), the Timed Up and Go (TUG) and the Get Up and Go (GUG) tests.

**Results:**

A total of 54 stroke survivors were included, with a mean age of 58.37 ± 12.42 years and a male predominance of 68.52%. The mean BBS score was 36.87 ± 14.34 with a minimum and a maximum of 10 and 56, respectively. Thirteen (24.07%) had balance impairments (BBS score ≤ 20), 34 (62.96%) had a TUG score ≥ 14 s (abnormal), 9 (16.67%) presented a moderate risk of falling and 6 (11.11%) presented high risk of fall with the GUG test. Post-stroke duration (odds ratio [OR] = 0.04; 95% CI: 0.04–0.30; *p* < 0.01), severity of disability (OR = 8.33; 95% CI: 1.03–67.14; *p* = 0.03) and the number of physiotherapy sessions (OR = 0.18; 95% CI: 0.03–0.93; *p* = 0.02) were significantly associated with balance impairments.

**Conclusion:**

Our results showed that almost one quarter of stroke survivors after discharge at the University Hospital of Parakou had balance impairments. Post-stroke duration, severity of disability and the number of physiotherapy sessions were significantly associated with balance impairments.

**Clinical implications:**

[AQ1] Balance should be regularly assessed in people post-stroke. Further studies should document the content of rehabilitation and any rehabilitative efforts to improve balance in people post-stroke in Benin.

## Introduction

Stroke is one of the leading causes of death and long-term disability worldwide and is the second leading cause of death behind cancers and myocardial infarction (Krishnamurthi et al. 2013). It has a devastating impact on the health of victims, their families and society (Mwaka 2019). Worldwide, 16 million new stroke cases are observed each year, accounting for 5.7 million deaths (Feigin et al. 2014). In Africa, the estimated prevalence rate of stroke is 3.5 cases per 1000 people, with an annual increase of 12% (Ezejimofor et al. 2016). Global estimates of stroke burden suggest that sub-Saharan Africa has the highest incidence (316 per 100 000 people per year), as well as prevalence (14.6 per 1000 people) and lethality (up to 43% at 1 month and 84% at 3 years) (Adoukonou et al. 2018, 2021; Owolabi et al. 2018). In Benin, the prevalence of stroke is estimated at 4.6 per 1000 in the urban population in Cotonou with hypertension as the most common risk factor, and this figure remains as the highest prevalence in the region (Cossi et al. 2012). Recently, a door-to-door community survey conducted in Parakou found an overall prevalence of stroke survivors of 1156 per 100 000 inhabitants (Adoukonou et al. 2020).

Stroke results in balance disorders and these directly affect walking performance and quality of life (Faria-Fortini et al. 2019; Khanittanuphong & Tipchatyotin 2017). Rehabilitation therapies have been reported to be effective for post-stroke balance impairment (Li et al. 2019). After a stroke, one of the main goals of rehabilitation is to promote independence in activities of daily living. An important determinant of performance in activities of daily living is standing balance, which again is an important predictor of functional recovery and the ability to walk (Kossi et al. 2019; Tyson et al. 2007). There are a number of reports on the prevalence of balance impairments following a stroke, with figures ranging from 16.7% (Chang et al. 2015) to as high as 83% (Abou et al. 2021; Cho & Kim 2020; Tyson et al. 2006).

Balance disorders have an impact not only on walking but also on all activities of daily life, including transfers, toileting, dressing, quality of life and mobility, and they increase the risk of falls (Arienti et al. 2019; Corbetta et al. 2015). Ursin et al. (2015) also show that balance impairment during the acute phase of stroke is a predictor of cognitive impairment 1 year after stroke (Ursin et al. 2015). Balance disorders are characterised by a short support time and differences in step length and slow walking speed.

Post-stroke balance impairment appears to be significantly influenced by stroke survivors’ age, gender and post-stroke duration (Phan et al. 2019; Vincent-Onabajo, Musa & Joseph 2018). Although several studies have been conducted in the domain of stroke rehabilitation in Benin (Kossi et al. 2016; Niama Natta et al. 2015, 2020), gaps do remain in understanding balance impairments and associated factors, their treatments and the optimal approach to delivering services to this population, especially in sub-Saharan countries like Benin.

As with many stroke outcomes (Burke et al. 2014; Kossi et al. 2019), there is the likelihood of contextual (environmental and clinical characteristics) differences in the prevalence of post-stroke balance impairment. For instance, balance impairment has been reported to be associated with age (Vincent-Onabajo et al. 2018) and severity of stroke (Tyson et al. 2006), and stroke severity is known to differ by race and ethnicity (Reeves et al. 2013). Furthermore, there is evidence that more profound negative outcomes are linked to the socio-economic status of people with stroke (Li et al. 2018; Yeh et al. 2017). Given the negative consequences of impaired balance after stroke, identifying the gravity of the post-stroke balance impairment, especially its prevalence, is critical. Similarly, information on associations between stroke survivors’ socio-demographic and clinical attributes such as balance impairments may assist in identifying those at risk and subsequently in providing appropriate and targeted interventions. Indeed, unawareness of the peculiar rehabilitation needs of the patients with stroke and resource constraints may be some reasons why until now, Benin hospitals have not been sufficiently equipped with the human and infrastructural resources required to meet patients with stroke special rehabilitation needs. Rehabilitation strategies that aim to improve post-stroke recovery outcomes require a thorough understanding of these major determinants. This is why, in our study we aimed to study the frequency and factors associated with balance impairments amongst stroke survivors at the University Hospital of Parakou in 2020.

## Method

Eligible participants were identified from the records of the Neurology and Physiotherapy Departments of the University Hospital of Parakou between 01 January 2020 and 30 September 2020, and the selected participants were recruited to our study.

The following conditions were verified before a patient was enrolled: age at least 18 years, diagnosed with a stroke in 2020, able to walk with or without assistance and no major cognitive impairments that may prevent him or her from clearly understanding instructions for tests (Brief Community Screening Instruments for Dementia in Primary Care score ≥ 6). A volunteer sampling approach was used to recruit participants. The minimum sample size was determined by considering 5% margin of error, 95% confidence level and 16% prevalence of balance impairments (Chang et al. 2015). The total sample size, 206, was proportionated to the five public and private hospitals in Parakou city based on their total number of beds. The minimum sample size required at the University Hospital of Parakou was then estimated at 49 participants. Patients who had other neurological impairments with permanent degenerative damage (e.g. Parkinson’s disease, Alzheimer disease, etc.) were excluded. Eligible participants were contacted by phone and invited to participate in our study. Then, an appointment for assessment was arranged. These assessments took place in August and September 2020.

### Outcomes

Assessments were undertaken by a physiotherapist with more than 10 years of experience in neurological rehabilitation. The primary outcome, balance impairments, was evaluated by using the Berg Balance Scale (BBS). The BBS is a psychometrically sound measure of balance impairment for use in post-stroke assessment (Blum & Korner-Bitensky 2008). However, given its floor and ceiling effects and its low sensitivity to detect risk of falling, it is advisable for clinicians to use the BBS in conjunction with other balance measures. These limitations of the BBS are understandable because falls are multifactorial and not related only to balance impairments. The Get Up and Go Test (GUG Test) and the Timed Up and Go Test (TUG) are more sensitive in detecting the risk of fall compared with the BBS (Blum & Korner-Bitensky 2008).

The risk of fall was assessed by using the GUG test (Mathias, Nayak & Isaacs 1986) and the TUG test (Podsiadlo & Richardson 1991). The GUG test assesses sitting–standing transfers, walking and position changes. With the participant sitting comfortably in an armchair 3 m from the wall, he is asked to stand up, stand for a few moments, head towards the wall, turn around without touching the wall, come back to the chair and sit.

This test is categorised into four levels (ranging from 1 = no fall risk to 4 = high fall risk). The GUG test is a valid, reliable and easy-to-administer clinical tool in people post-stroke (Chan et al. 2017; Piva et al. 2004).

The TUG test measures in seconds the time required for the patient to complete the GUG test. Time less than 14 s was considered ‘Normal’, and time equal or greater than 14 s was considered ‘Abnormal’, meaning that the patient needs more time than usual to complete the GUG test. The TUG test is valid with excellent test–retest reliability and good responsiveness in people post-stroke (Alghadir et al. 2018; Podsiadlo & Richardson 1991). Evaluations took place at the neurorehabilitation unit. For each patient, all assessments were completed in one session.

The modified Rankin Scale (mRS), which is one of the most widely used clinician-reported tools, was used to categorise overall disability for each participant. Participants were rated from 0 (‘no symptoms at all’) to 5 (‘severe disability’). High mRS scores indicate worsening status. Validated for use in patients with stroke (Rankin 1957), the mRS seems to be more reliable, sensitive and responsive compared with the Barthel Index in measuring stroke disability (Balu 2009; Banks & Marotta 2007).

Information about factors that may be associated with balance was collected by a general questionnaire. These factors included socio-demographic characteristics, clinical and rehabilitation information (frequency and the total number of sessions) and were selected based on the literature (Blum & Korner-Bitensky 2008; Vincent-Onabajo et al. 2018) and also by consultation with experts in stroke rehabilitation in our laboratory. Time since stroke was recorded as time since stroke onset and the date of evaluation.

### Data analysis

Data analysis was performed by using Epi Info 7.2.2.6 software. Nominal and ordinal variables are presented as proportions and quantitative variables as means with standard deviation. Bivariate analyses with Fisher’s exact tests were used to investigate the association between socio-demographic, clinical and balance impairments through the odds ratio (OR) with its 95% confidence interval. The significance threshold for all statistical analyses was set at < 0.05.

### Ethical considerations

This was a cross-sectional study with an analytical focus that received approval from the local ethics committee of the University Hospital of Borgou, Parakou/Benin under the number: 1435/20/MS/DC/DDS-B/CHUD-B-A/SAAE/DGAP. Our clinical study was conducted with authorisation from the hospital institution, and informed consent was obtained from the patients. This is the current regulation in the Republic of Benin. Participants accepted their agreement to participate in our study by signing a consent form.

## Results

### Characteristics of study participants

**FIGURE 1:**
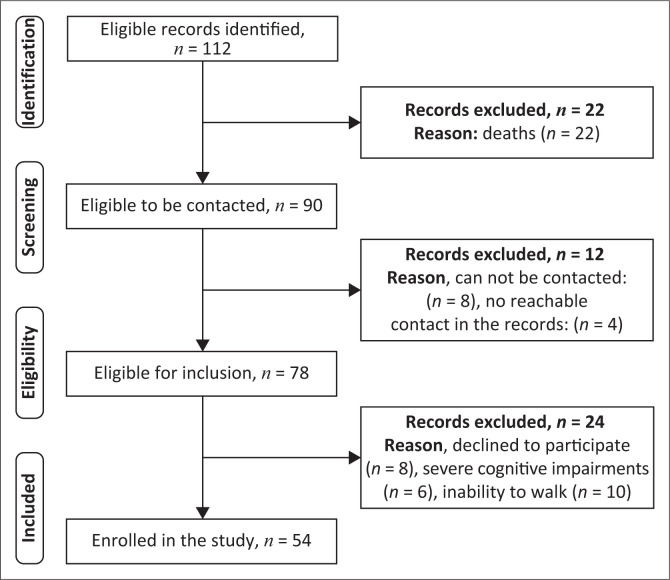
Flow chart of eligibility and inclusion.

A total of 112 eligible patients were identified in the records. The process by which study participants were included is shown in Figure 1. The mean age of the included participants was 58.37 years (± 12.42) with a minimum and a maximum of 32 and 86 years, respectively, and a male predominance of 68.52%. Socio-demographic and clinical characteristics of the participants are described in Table 1.

**TABLE 1:** Socio-demographic and clinical characteristics of the participants (*n* = 54).

Characteristics	Sample	
	*n*	%
**Socio-demographic**		
**Age (year)**		
32–45	10	18.52
46–55	12	22.22
56–65	15	27.78
66–75	13	24.07
76–86	4	7.41
Sex		
Male	37	65.52
Female	17	34.48
**Religion**		
Christian	35	64.81
Muslim	18	33.33
Other	1	1.86
**Life style**		
Lives alone	3	5.55
As couple	51	94.45
**Occupation**		
Housewife	8	14.82
Official	8	14.82
Artisan	11	20.37
Trader	11	20.37
Retired	13	24.07
Farmer	3	5.55
**Clinical**		
**Type of stroke**		
Ischaemic	34	62.96
Haemorrhagic	16	29.63
Undetermined	4	7.41
**Cerebral hemisphere injured**		
Right	26	48.15
Left	28	51.85
**Post-stroke duration,** month		
≤ 1	8	14.82
1–6	34	62.96
≥ 6	12	22.22
**Severity of disability**		
No symptoms	6	11.11
No significant disability despite symptoms	16	29.63
Light disability	12	22.22
Moderate disability	7	12.96
Moderately severe disability	13	24.08
**Number of sessions per week**		
One	6	11.11
Two	22	40.74
Three	26	48.15
**Number of sessions already completed**		
< 9	28	51.85
9–18	22	40.74
> 18	4	7.41

### Risk of fall amongst participants

The mean BBS score was 36.87 ± 14.34 with a minimum and a maximum of 10 and 56, respectively. Of the 54 participants, 13 (24.07%) had balance impairments (BBS score ≤ 20) and 34 (62.96) had a TUG score ≥ 14 s (abnormal). Using the GUG test, 9 (16.67%) participants presented with a moderate risk of falling whilst 6 (11.11%) presented with a high risk of falling. Table 2 illustrates the distribution of study participants according to their balance status.

**TABLE 2:** Distribution of study participants according to their balance status with the Berg Balance Scale, the Timed Up and Go test and the Get Up and Go test (*n* = 54).

Variables	*N*	%
**BBS score**		
Good balance (score ≤ 20)	23	42.59
Balance acceptable (20 < score ≤ 41)	18	33.33
Balance disorders (41 < score ≤ 56)	13	24.07
**Timed Up and Go test**		
Normal (score < 14 s)	20	37.04
Abnormal (score ≥ 14 s)	34	62.96
**Get Up and Go test**		
No risk of fall	18	33.33
Low risk of fall	21	38.89
Moderate risk of fall	9	16.67
High risk of fall	6	11.11

BBS, Berg Balance Scale.

### Association between balance impairments and socio-demographic factors

Table 3 presents the association between balance impairments and socio-demographic factors by using the BBS score. No socio-demographic variables were significantly associated with balance impairments in our sample.

**TABLE 3:** Association between balance impairments and socio-demographic factors by using the Berg Balance Scale score (*n* = 54).

Variables	Balance impairments based on the BBS score		OR	95% CI	*p*
	*N*	%			
**Age (years)**					
32–45	3	30.00	1	-	-
46–55	1	8.33	0.21	0.02–2.47	0.19
56–65	1	6.67	0.17	0.02–1.91	0.12
66–75	5	38.46	1.47	0.25–8.43	0.67
76–86	3	75.00	0.78	0.06–10.46	0.87
**Sex**					
Male	10	27.03	1	-	-
Female	3	17.65	0.58	0.14–2.45	0.45
**Religion**					
Christian	6	17.14	1	-	-
Muslim	7	38.89	1.88	0.54–6.49	0.31
Other	0	0.00	-	-	-
**Life style**					
Lives alone	1	33.33	1	-	-
In couple	12	23.53	0.70	0.13–3.76	-
**Occupation**					
Housewife	2	25.00	1	-	-
Official	1	12.50	0.43	0.03–5.99	0.52
Artisan	3	27.27	1.13	0.4–8.99	0.91
Trader	2	18.18	0.67	0.07–6.11	0.72
Retired	5	38.46	1.88	0.27–13.20	0.53
Farmer	0	0.00	-	-	-

OR, odds ratio; CI, confidence interval; BBS, Berg Balance Scale.

### Association between balance impairments and clinical characteristics of study participants

Table 4 shows the distribution and the association between balance impairments and clinical characteristics of participants by using the BBS score. Thirty-four (62.96%) of the stroke cases were ischaemic and 29.63% of the stroke cases were haemorrhagic. Undetermined strokes accounted for 7.41% of patients. The left cerebral hemisphere was injured in 51.85% of stroke survivors.

**TABLE 4:** Balance impairment and stroke characteristics (*n* = 54).

Variables	Balance impairments based on the BBS score		OR	95% CI	*p*
	*N*	%			
**Type of stroke**					
Ischaemic	8	23.53	1	-	-
Haemorrhagic	4	25.00	2.03	0.52–7.99	0.31
Undetermined	1	25.00	1.08	0.09–11.92	0.95
**Cerebral hemisphere injured**					
Right	8	30.77	1	-	-
Left	5	17.86	2.04	0.57–7.33	0.27
**Post-stroke duration,** month					
≤ 1	6	75.00	1		
1–6	4	11.76	0.04	0.01–0.30	< 0.01*
≥ 6	3	25.00	0.11	0.01–0.87	0.03*
**Severity of disability**					
No symptoms	0	0.00	-	-	-
No significant disability despite symptoms	0	0.00	-	-	-
Light disability	0	0.00	-	-	-
Moderate disability	2	22.22	1	-	-
Severe disability	10	76.92	8.33	1.03–67.14	0.03*

*, statistically significant.

OR, odds ratio; CI, confidence interval; BBS, Berg Balance Scale.

The average post-stroke duration was 3.14 ± 2.69 months. More than three-quarters of our sample had a post-stroke duration of 1–6 months. Stroke duration was significantly associated with balance impairments with a decreasing ratio of 0.96 between 1–6 months and ≤ 1 month (OR = 0.04; 95% CI: 0.01–0.30; *p* < 0.01) and a ratio of 0.89 between ≥ 6 months and 1–6 months (OR = 0.11; 95% CI: 0.01–0.87; *p* = 0.03). In terms of severity of stroke, 12.96% of survivors had a moderate disability whilst 24.08% of survivors had a moderately severe disability. Stroke severity was significantly associated with balance impairment with an OR = 8.33; 95% CI: 1.03–67.14; *p* = 0.03.

### Balance impairments and rehabilitation-related factors

Table 5 shows the distribution and the association between balance impairments according to rehabilitation-related factors. Of the 54 stroke survivors, 48.15% of patients had 3 sessions per week and 57.41% of patients had fewer than 10 sessions since admission. Rehabilitation was significantly associated with balance impairment, with a decreasing ratio of 0.10 between those who had < 10 rehabilitation (physiotherapy) sessions compared with those who had ≥ 10 sessions (OR = 0.09; 95% CI: 0.01–0.74; *p* < 0.01).

**TABLE 5:** Balance impairments and rehabilitation-related factors (*n* = 54).

Variables	Balance impairment based on the BBS score		OR	95% CI	*p*
	*N*	%			
**Number of sessions per week**					
One	0	0.00	-	-	-
Two	4	16.00	1	-	-
Three	9	34.62	1.75	0.47–6.48	0.40
**Number of sessions already completed**					
< 9	10	35.72	1	-	-
9–18	3	13.64	0.18	0.03–0.93	0.02*
> 18	0	0.00	-	-	-

*, statistically significant.

OR, odds ratio; CI, confidence interval; BBS, Berg Balance Scale.

## Discussion

The purpose of our study was to determine the frequency and factors associated with balance impairments amongst stroke survivors post-discharge at the University Hospital of Parakou in northern Benin in 2020. In our study, 24.07% of participants had balance impairments.

Post-stroke duration, severity of disability and the number of physiotherapy sessions were significantly associated with balance impairments.

Recently, Vincent-Onabajo and Joseph (2018) examined the prevalence and factors associated with balance impairments after stroke in Nigeria. They found stroke survivors’ balance impairment to be significantly influenced by post-stroke duration. This result is similar to ours.

The days following a stroke are commonly a phase in which many of the consequences of the impairment are fully manifested (De Haart et al. 2004). It is therefore not surprising that in this phase, particularly in the acute phase (< 1 month), stroke survivors are likely to present more balance impairments than those in the chronic phase (> 6 months) (Karthikbabu et al. 2018; Rose et al. 2017). Several studies agree that motor recovery is more consistent during the subacute phase (4 to 15 weeks) and that subsequently there is a plateau from 6 months after the stroke (Schaechter 2004). However, although recovery is slower at 6 months after the stroke, different studies have also shown possible improvement up to several years later (Langhorne et al. 2011; Murphy 2009). The possibility of recovering beyond a plateau may be because of the fact that intensive therapy oriented to the recovery of function focusses on obtaining an improvement whatever be the post-stroke duration (Mweshi et al. 2016).

Fall prevention is an important clinical consideration in ambulatory post-stroke survivors and a recovery of balance is essential for independence in daily living (Pollock, Eng & Garland 2011). We found a significant association between the number of rehabilitation sessions undertaken by patients and balance impairments. Of the 13 stroke survivors with balance impairments, nine patients had three sessions per week. Unfortunately, 10 out of the 13 had fewer than 9 sessions of physiotherapy from onset to enrolment. In other words, most of the participants had not followed more than 3 weeks of rehabilitation. In fact, post-stroke recovery strongly depends on several parameters including the frequency of sessions per week, the intensity (dose) of exercises, the duration of each sessions, the total volume of interventions and also the type of rehabilitation exercise implemented (Corbetta et al. 2015). In particular, improvement of balance has been shown to be influenced by training intensity, with frequencies of at least five sessions a week of 30–60 min each producing significant improvements within 4 weeks (Nindorera et al. 2021). Longer-term interventions (e.g. 12 weeks) appear to be needed to reduce the severity of motor impairments (Daly et al. 2011; Globas et al. 2012). A recent systematic review and meta-analysis on balance impairments after stroke concluded that functional task-training associated with musculoskeletal intervention and/or cardiopulmonary intervention and sensory interventions seem to be immediately effective in improving balance and postural stability, respectively (Hugues et al. 2019). Another systematic review with meta-analysis demonstrated the evidence of the effectiveness of robot-assisted therapy to produce significantly positive improvements in balance function amongst patients with stroke compared with those not using this method (Zheng et al. 2019).

Our results show that the severity of the disability is significantly associated with balance impairments in stroke survivors. These results are similar to the results of Tyson et al. (2006), who also found that patients with severe balance disability had severe disabilities. Our results could also be explained by the fact that most of the patients we included were in the subacute phase, a phase in which motor impairment is the main symptom; muscle weakness and loss of voluntary movement are common problems that occur immediately after a stroke and contribute to a reduction in walking performance during the subacute stage (Wonsetler & Bowden 2017).

### Study strengths and limitations

To our knowledge, our study is the first to explore the factors associated with balance impairments in a sample of Beninese stroke survivors. This is a first step that may help to establish better strategies for the rehabilitation of this population (systematic assessment of balance disorders, an efficient platform for the best rehabilitation care of patients with stroke, etc.).

However, our findings need to be interpreted in the context of some potential limitations. Indeed, it has been shown that the BBS, used as a primary outcome measure in our study, presents floor and ceiling effects. As a consequence, the frequency of balance impairments may have been under- or over-estimated. However, this limitation could be minimised by the use of the GUG and TUG tests in conjunction with the BBS.

## Conclusion

Our findings are a valuable contribution to evidence on the balance impairments in patients with stroke in Benin. Our results show that the frequency of balance impairments amongst patients with stroke at the University Hospital of Parakou is 24.07%. Our findings also show that post-stroke duration, the severity of disability and the number of physiotherapy sessions are significantly associated with balance impairments. Further studies should document the content of rehabilitation and any rehabilitative efforts to improve balance in people post-stroke in Benin.
